# Cell Permeable Peptides: A Promising Tool to Deliver Neuroprotective Agents in the Brain

**DOI:** 10.3390/ph3020379

**Published:** 2010-02-03

**Authors:** Xanthi Antoniou, Tiziana Borsello

**Affiliations:** Istituto di Ricerche Farmacologiche "Mario Negri", Via La Masa 19, 20157 Milano, Italy; E-Mail: xanthi.antoniou@marionegri.it (X.A.)

**Keywords:** CPPs, neuroprotection, protein-protein interaction, drug discovery, brain

## Abstract

The inability of most drugs to cross the blood-brain barrier and/or plasma membrane limits their use for biomedical applications in the brain. Cell Permeable Peptides (CPPs) overcome this problem and are effective *in vivo*, crossing the plasma membrane and the blood-brain barrier. CPPs deliver a wide variety of compounds intracellularly in an active form. In fact, many bioactive cargoes have neuroprotective properties, and due to their ability to block protein-protein interactions, offer exciting perspectives in the clinical setting. In this review we give an overview of the Cell Permeable Peptides strategy to deliver neuroprotectants against neurodegeneration in the CNS.

## 1. Introduction

The discovery of new drugs to treat diseases of the central nervous system (CNS) is one of the most challenging future objectives. There are two fundamental difficulties in the delivery of drugs to the CNS. The first is to cross the blood brain barrier (BBB), and the second is to specifically target neurons within the brain. Methods such as microinjection, electroporation or gene-gun of viral vectors are associated with low transfer efficiency and damage to membrane integrity. Additionally these methods often led to an immunoresponse [1]. New cargo strategies with improved delivery in neuronal cells *in vitro* and *in vivo* would have almost unlimited application.

In the last decade drug technology has developed the so-called Cell Permeable Peptides (CPPs). The approach is currently a major avenue in engineering non-invasive delivery systems. The large number of different cargo molecules that have been efficiently delivered by CPPs ranges from small peptides to proteins, siRNA [2], small therapeutical molecules [3], and even liposomes and particles [4].

CPPs constitute very promising tools. In fact three CPPs have progressed to clinical testing in humans [5,6]. However, the potential of CPPs in drug development is not yet well explored. There exist some important limitations of this methodology as well as some problems in the interpretation of results obtained from studies in the field. Subsequently many efforts are concentrated in demonstrating the value of these peptides for target validation, cell-based efficacy with novel compounds and a greater understanding of biological mechanisms. Focused studies on *in vivo* pharmacokinetics, tissue distribution, metabolism and toxicology of CPPs are needed to evaluate the use of these compounds for novel therapeutics. Still, considering the difficulties that conventional methods have to reach intracellular or nuclear targets, the use of CPPs in gene therapy, cancer and neurodegenerative treatments are of great interest [7].

### 1.1. Cell Permeable Peptides

CPPs are short positively charged peptides composed of basic residues (lysine or arginine) of 20 to about 50 amino acids which can cross the cellular plasma membrane [8] and efficiently deliver biological active proteins to all tissues, including the brain [9,10,11]. The intracellular delivery of bioactive molecules takes place by employing membrane-permeable carrier peptide vectors, the most common ones being Tat, Antp-HD, and related arginine-rich sequences. Remarkably, advancement of technology led to the production of CPPs that can enter specifically the mitochondria, better known as the SS (Szeto-Scchiller) peptides [12].

**Figure 1:**
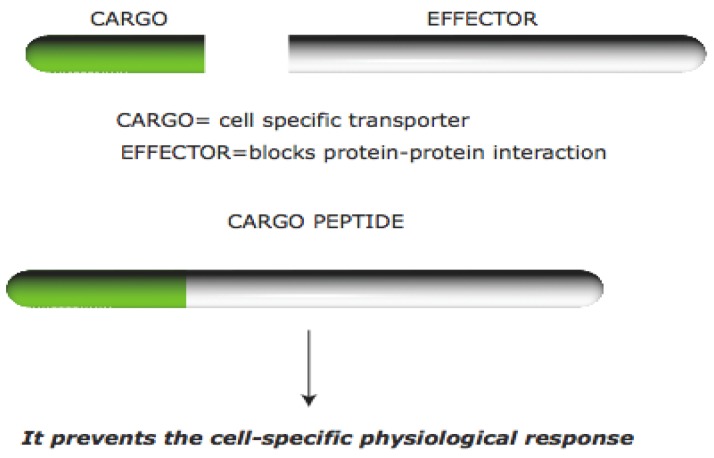
Schematic representation of a typical Cell Permeable Peptide. CPPs are composed of a Cargo molecule (TAT-green) that allows cell penetration and an Effector (white) molecule that interferes with a specific protein interaction. This peptide results in a bioactive cargo that is able to interfere with a cellular physiological response.

The typical structure of a CPP is composed of a CARGO peptide linked to an effector-peptide. The first peptide allows intracellular penetration while the second peptide interferes with a key protein-protein interaction and results in a physiological response as shown in Figure 1. The CPPs may be synthetically designed constructs and allow non-invasive import of different cargos. 

This technology shows numerous advantages that include (a) high and rapid output, (b) relatively low costs for synthesis, (c) versatility: few scaffold cores can be used to generate several compounds able to act at different levels from kinases inhibitors to secretase-like molecules, (d) stability (when synthesized in D-amino acids), low catabolism; and (e) low toxicity. Last but not least CPPs can be active in a range of nanomolar concentrations [14,15]. Consequently, CPPs are attractive drug delivery tools being capable of targeting intracellular signalling pathways as well as interfering with intracellular protein-protein complexes to rebalance a perturbed cellular function. 

However CPPs present also some limitations. Today, the mechanisms by which CPPs penetrate the cell membrane are not quite understood. Penetration differs depending on the cell line, tissue but also on the CPP used as well as on the concentration [16,17,18]. Most importantly, the *in vivo* CPP’s toxicity and immunogenicity are largely unexplored. 

### 1.2. Protein–Protein Interactions in the Brain

Protein–protein interactions modulate the neuronal biological response to external stimuli and neuronal death. A huge body of information about signaling pathways in neurodegeneration and neuronal death allows identification of new targets to interfere with neurodegenerative mechanisms. Of those NMDA receptor pathways, Mitogen Activated Protein Kinases (MAPKs) and apoptotic pathways have been extensively studied [19,20,21]. CPPs inhibit protein–protein interactions and provide a new and alternative tool to modulate signaling pathways in the brain. Furthermore the development of more sophisticated computational methods allows a detailed analysis of protein-protein interactions and consequently the design of improved inhibitors with higher affinity. We thus believe that synthetic peptides may represent the tool to prevent neurodegeneration in the adult central nervous system.

The CARGO-strategy can be used to hinder protein-protein interactions between an enzyme and its substrate, between a catalytic domain and its regulatory domains, and also between protein motifs and scaffolding domains. The most common way to interfere with protein-protein interactions is by disruption of protein-protein interactions [22]. However it is also possible to interfere with a signaling pathway by stabilization of protein-protein interactions [23] or by disruption of intra-protein contacts during folding [24].

Some molecular targets have been identified as key mediators in neuronal death or neurodegenerative processes and we will discuss here the application of the designed CPPs against these modulators.

## 2. Neuroprotective CPPs

### 2.1. D-JNKI1

JNK is a member of the MAPK family with an important role in excitotoxic mechanisms. The D-JNKI1 peptide (that is the D-retro-inverso form, made of aminoacids in reversed sequence order) has been engineered by linking the 20 amino acid JNK-inhibitory sequence of IB1/JIP-1 scaffold protein (JBD_20_) to the 10 amino acid HIV- transporter sequence [25]. JIP-1 scaffold protein and c-Jun, the main target of JNK, share a similar JNK binding motif. However the JNK binding affinity to JIP-1 is about 100-fold higher than to c-Jun. Barr and collaborators found that a shorter peptide sequence (RPKRPTTLNLF = TI-JIP) based on amino acids 143–153 on the JBD of JIP-1, partially overlapping with the sequence described by Bonny [25] was also able to prevent c-Jun phosphorylation [26].

**Figure 2:**
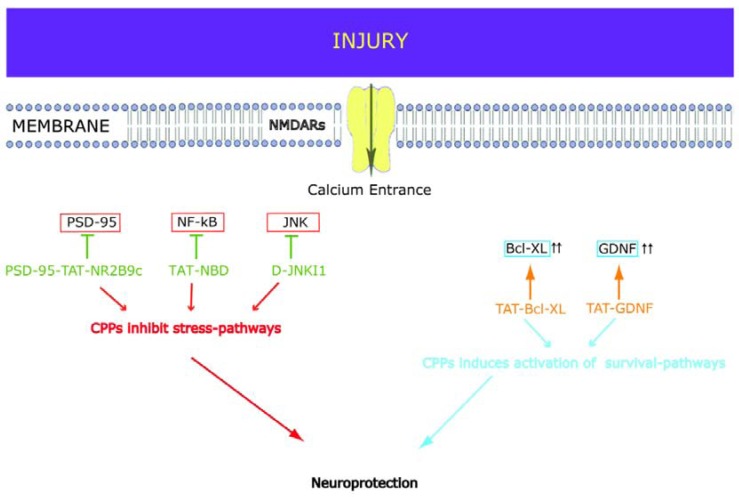
Schematic representation of the described neuroprotective CPPs and their protein targets. The stress pathways that are triggered by injury and the survival pathways that are activated in neuroprotective mechanisms are presented. We here indicate the two different families of CPPs: inhibitor (⊥) CPPs in red and stabiliser (⇑⇑) CPPs of protective proteins in orange. Inhibitor-CPPs: PSD-95-TAT-NR2B9c blocks the interaction between PSD-95 and the NMDA receptor subunit NR2B. D-JNKI1 blocks the association of JNK with its JBD-targets and inhibits JNK signaling pathways. TAT-NBD inhibits NF-kB activity by blocking association of its key regulators: NEMO (NF-kB essential modulator) with the IKKs (I Kappa B kinases). Stabiliser-CPPs: TAT-Bcl-xL induces Bcl-xL overexpression and subsequently prevents caspase activation. TAT-GDNF induces GDNF stabilization and inhibits caspase activation.

The inhibitory action of the D-JNKI1 peptide is fundamentally different from that of classical small chemical inhibitors [27,28]. D-JNKI1 does not inhibit JNK activity but modulates JNK allosterically and selectively blocks access to its substrates by a competitive mechanism [25,27,28] (Figure 2). To determine the specificity of the peptide in blocking JNK action, we characterized its effects on the activity of 40 different kinases (10 µM peptide, 10 µM ATP) towards their respective substrates in a cell free system. It did not interfere with the activities of other kinases [27,28] proving its exceptional superiority. 

D-JNKI1 prevents both apoptosis [25] and necrosis [29] *in vitro* and is a potent neuroprotectant against both transient and permanent ischemia *in vivo* [10,28,30,31]. More in detail, we obtained strong protection in two models of middle cerebral artery occlusion (MCAO): transient occlusion in adult mice and permanent occlusion in 14-days-old rat pups. In the first model, intraventricular administration 6 h after occlusion reduced the lesion volume by more than 90% for at least 14 d while in the second model systemic delivery reduced the lesion by 78% and 49% at 6 and 12 h after ischemia, respectively. D-JNKI1 is also protective against toxic drug and acoustic trauma induced auditory hair cell death [32], against retinal ganglion cells death following optic nerve crush [33] and Traumatic Brain Injury [33]. More recently different authors proved that D-JNKI1 is protective against spinal nerve ligation [34], viral encephalitis [35], myocardial ischemia-reperfusion injury [35], and in an Alzheimer’s disease (AD) *in vitro* model where it prevented Aβ fragment production by reducing the rate of amyloidogenic processing in favour of the non-amyloidogenic one [35]. Although most applications indicate D-JNKI1 as a potent neuroprotector, the peptide was initially described as a blocker of β-cell death in an *in vitro* model of inflammation [25]. A more recent report indicated the potential use of D-JNKI1 in liver cancer [36], while Lehnert *et al*. [37] reported that D-JNKI1 application blunts hepatic damage after hemorrhagic shock and before resuscitation. These data show the multifaceted protective actions of D-JNKI1 and suggest that it can offer interesting possibilities for therapeutic application in preventing neuronal loss and more generally cell death.

### 2.2. PSD-95-Tat-NR2B9c

In a similar manner a TAT peptide that interferes with the NMDA receptor (NMDAR) and the postsynaptic density 95 (PSD-95) protein has been synthesized and tested successfully in models of excitotoxicity *in vitro*, as well as in *in vivo* models of permanent and transient focal ischemia [38,39,40], (Figure 2). The success of the PSD-95-TAT-NR2B9c lies on its ability to dissociate NMDA-receptors from the neurotoxic signaling pathways (Figure 2) but it does not block normal synaptic activity or calcium entrance. The authors proved that intravenous injection of the peptide 3 hours after injury attenuated infarct size by 50%, and most importantly improved neurological function in a stroke model of transient middle cerebral artery occlusion [38]. The PSD-95-TAT-NR2B9c was also efficient in attenuating brain injury in an *in vivo* model of epilepsy [41] as well as in chronic pain inflammation [42]. The results indicate that disruption of the PSD-95-NMDAR interaction can represent a potential therapeutic approach in brain injury.

### 2.3. NF-kB

NF-kB is a well-characterized transcription factor with a key role in inflammation as well as cell death and survival. Since the outcome of ischemic brain damage is determined by interactions between excitotoxic, inflammatory and apoptotic pathways, and NF-kB signaling interferes with all these processes its impact in ischemia has been extensively investigated [43]. 

The contribution of NF-kB varies in different models of neurotoxicity. Nevertheless inhibitors that block NF-kB signaling have been successful and have opened the road towards more successful therapeutical strategies in several disease models [44,45]. A number of NF-kB CPP inhibitors have been reported (for an extensive review see Orange *et al*. [46]). The TAT-NEMO binding domain (TAT-NBD) peptide inhibits NF-kB activity by blocking association of its key regulators, namely the NEMO (NF-kB essential modulator) with the IKKs (I Kappa B kinases) (Figure 2). Inhibition of NF-kB activity by the TAT-NBD peptide prevented p53 upregulation and accumulation, mitochondrial cytochrome-c release and activation of caspase-3 and reduced brain damage by more than 80% in an *in vivo* model of neonatal cerebral hypoxia-ischemia. Most importantly administration of TAT-NBD had a therapeutic window of at least 6 hours, an important finding with practical applications. The authors could further show that TAT-NBD by inhibiting NF-kB could increase survival in a neuronal cell line, indicating that the peptide acts directly on neurons [44]. 

The above data suggest that future application of NF-kB CPP inhibitors in treatment of neonatal ischemia is promising. However, as suggested by the authors [45], timing and duration of NF-kB inhibition will be determining factors for the success of NF-kB CPPs. 

### 2.4. GDNF

The glial line-derived neurotrophic factor (GDNF) is a potent neurotrophic factor and an established neuroprotector in various models of cerebral ischemia and has been long identified as another attractive pharmacological target. Similar to other neurotrophic factors, GDNF does not pass the BBB. In order to ensure tissue accessibility, a TAT-GDNF fusion protein was synthesized and tested in models of cerebral ischemia. Intravenous administration of TAT-GDNF diminished brain injury following both mild and severe ischemic insults [47]. More importantly, recovery was achieved even when the TAT fusion proteins were administered after the ischemic insult. The TAT-GDNF peptide was additionally tested in a model of optic nerve axotomy [48]. In all models the TAT-GDNF peptide attenuated caspase-3 expression (Figure 2).

### 2.5. Bcl-xL

In a similar manner the Bcl-2 family proteins play a critical role in ischemic neuronal death. In particular Bcl-2 and Bcl-xL are anti-apoptotic proteins that contribute to neuronal survival. Bcl-xL exerts its functions via direct interaction with caspases but also by inhibiting the release of pro-apoptotic factors, such as cytochrome c from the mitochondria [13,49], (Figure 2). In models of cerebral ischemia expression of Bcl-xL is markedly decreased in dying neurons but is sustained in surviving ones [50,51]. The contribution of Bcl-xL in both necrotic and apoptotic pathways renders it an attractive therapeutical target in cerebral ischemia and in CNS disorders as a whole. 

In line with the above a TAT-Bcl-xL fusion protein has been engineered and tested for its neuroprotective effects both in *in vitro* and *in vivo* models [52]. Similarly TAT-GDNF intravenous delivery of the TAT-Bcl-xL peptide was successful in a mouse model of transient focal cerebral ischemia (*in vivo*). Furthermore, delivery of the TAT-Bcl-xL peptide protected neurons from death in an *in vitro* and *in vivo* model of Parkinson’s disease (PD) providing some of the first evidence that CPPs can be used in models of chronic neurodegenerative diseases [53,54,55]. 

## 3. CPPs in Chronic Neurodegenerative Disorders

Many key modulator pathways in neurodegeneration have been identified and the use of CPPs that interfere with these key players may represent novel therapeutical hope (see Figure 2). Indeed, the current findings in the field of CPPs in acute neurodegenerative disorders are fascinating [28,46,47,54,56]. The next step will be to test the application of CPPs in chronic diseases. To our knowledge, only few CPPs have been designed and tested in models of chronic neurodegenerative disease, namely AD [57], PD [55] and amyotrophic lateral sclerosis (ALS) [58]. 

The first, Bcl-xL peptide protected neuroblastoma cells from 1-methyl-4-phenylpyridinium, a selective neurotoxin. Additionally systemic application of the peptide in aged mice protected dopaminergic neurons following administration of MPTP [55]. The second, a TAT peptide coupled to acetyltransferase, an enzyme implicated in acetylcholine synthesis and subsequently in the normal functioning of cholinergic neurons, improved long-term and spatial memory in an AD mouse model [57]. The TAT-NBD peptide was also tested in a chronic inflammation model of experimental allergic encephalomyelitis (EAE) [59]. In this model the peptide was injected for more than 50 days (every other day injections) without any signs of toxicity, and reduced EAE clinical symptoms.

Last but not least, the more recent characterization of peptides that specifically target the mitochondria and thus inhibit the formation of reactive oxygen species gave hope in the field of neurodegenerative disorders and not only. The so-called SS peptides have been tested successfully in animal models of PD and ALS [12,60]. Such data underline the possible use of CPPs in chronic neurodegenerative disorders. However the application of CPPs in chronic model of disease needs to be better validated. The major limitations of CPPs will be discussed in the section below.

## 4. Limitations and Pitfalls

There are four different limitations in CPP application as pharmaceutical tools: (a) the delivery strategy: tissue/cell specificity, (b) toxicity, (c) stability and (d) immunogenicity. 

### 4.1. Delivery Strategies: Tissue/Cell Specificity

*In vivo* CPPs penetrate most tissue types, a great limitation for clinical applications since injection of a CPP enzyme inhibitor will prevent the action of the target/enzyme systemically and can lead to unwanted side-effects. We can foresee two possible solutions to overcome this issue: (1) target tissue specific forms of an enzyme and (2) targeting of a specific cell feature, such as cell-binding ligands (e.g., receptors or antibodies) to subsequently render the uptake specific to a cell type [61]. Such an approach was recently tested by Kumar *et al*. [62] who showed that a small peptide consisting of the rabies virus glycoprotein (RVG) plus nine arginines allows transvascular delivery of siRNA in the CNS. Treatment with RVG-9R bound siRNA protected mice against fatal viral encephalitis [62]. This is to our opinion a fascinating approach that is based on the ability of the virus to recognize the acetylcholine receptor in the neurons, in combination to the ability of the short, positively charged 9R peptide to penetrate the cell. As reported by the authors, the efficacy of the peptide was relatively low (50%) and methods to improve delivery are required. Nevertheless repeated injection of the peptide did not induce inflammation in these mice, a promising finding for future clinical applications.

### 4.2. Toxicity

CPPs have been successful due to their ability to internalize within cells. The internalization process could potentially alter the cell homeostasis and thus has to be properly characterized. Although most of the studies described in the literature would suggest that in low concentrations CPPs are not toxic, some reports have raised doubts. Cationic peptides showed toxicity effects on rat neuronal cultures [63] as well as other *in vitro* models [56,64]. 

Toxicity may be partially related to the length of the peptide [65]. A detailed study by Cardozo *et al*. [16] provided evidence that Tat is significantly less toxic than Antp in five cell lines tested. Additionally some studies proved that the TAT long sequences 48–57 were not significantly toxic compared to penetratin [56,64,66]. Overall a lesson that we can learn from these studies is that CPPs should be designed with the highest affinity for their intracellular targets, but also with the shortest possible sequence. By doing so, CPPs can be used in low concentrations thus lowering the risk of toxicity.

A few reports of safety studies with Tat exist, but there are no comparable examples with other CPPs. Future reports should be comparative and examine tissue pathology, blood-based markers of liver or kidney function, blood pressure, heart rate, respiratory function and animal behavior. Safety will be a critical factor in distinguishing the superiority of one CPP over another.

### 4.3. Stability

The stability of CPPs is important for *in vivo* application*.* Only few studies have investigated the cellular metabolism of CPPs until now [65,67,68]. Increased stabilization and thus longer lasting biological activities of CPPs can be achieved by using retro-inverso (D-) enantiomers that are less sensitive to protease degradation. Such an approach has been successful and has been documented in the case of D-JNKI1 [28]. An alternative is to design protecting groups for metabolically labile sites, as well as PEGylation of compounds. The latter however have not been extensively studied and may result in toxicity [69]. One could even speculate that the short life of the CPPs can be an advantage in some cases and resolve the potential toxicity of a long-term inhibition obtained with a long-life of the CPP. In fact, the use of CPPs against acute brain injury did not require a long life of the compound. 

### 4.4. Immunogenicity

The immunogenicity and immunotoxicity of CPPs is poorly documented. Still, it is unlikely that CPPs, with a short plasma half-life and rapid tissue distribution, will generate an immune response by intravenous administration. Nevertheless we should also not exclude the possibility that CPPs derived from non-human proteins can potentially induce an immune response especially in therapies targeting chronic neurodegenerative disorders.

## 5. Conclusions

Several obstacles have to be overcome in order to design successful pharmacological strategies based on CPPs. Preclinical and pharmacological data on the already available peptides are scarce. One should bear in mind that species specificity can have a determinant role and further studies are definitely required to explore this matter.

The development of efficient CPPs able to target the right organs, cells and cellular compartments is of high priority. Better understanding of the plasma membrane properties will hopefully provide important information to develop original strategies for more efficient penetration. Deciphering of signaling mechanisms will have consequences in the understanding of several neuropathologies but also in designing the right approaches. Combined therapies with more than one CPP, targeting more than one signaling pathway may increase clinical outcome.
